# Effects of carbohydrate intake and body composition on short-distance swimming performance in adolescents

**DOI:** 10.1080/15502783.2025.2550201

**Published:** 2025-09-16

**Authors:** Larissa de Brito Medeiros, Maria Rayssa Silva do Nascimento, Hellenrita Lima da Luz Castro, José Atson da Silva Barbosa, Rafael Ikaro de Souza Carvalho

**Affiliations:** João Pessoa University Center – UNIPÊ, Nutrition Program, Brazil

**Keywords:** Swim time, body fat percentage, energy availability, Nutrition

## Abstract

**Background:**

This study aimed to evaluate the influence of carbohydrate intake and body composition on athletic performance in adolescent swimmers

**Methods:**

A cross-sectional study was conducted with 22 adolescent swimmers (both sexes) from a sports center in Paraíba, Brazil. Pre- and post-training carbohydrate intake was assessed using 24-hour dietary recalls and compared against the International Society of Sports Nutrition (ISSN) guidelines. Body composition was estimated via skinfold measurements, using age- and sex-specific cutoff points. Swimming performance was assessed based on the time to complete a 25-meter front crawl. Performance was categorized as optimal ( < 15 s), good (15–16 s), or regular ( > 16 s). These thresholds were defined by the authors based on the distribution of swim times in the study sample. Statistical analysis was performed using IBM® SPSS® Statistics 20.0. Associations between categorical variables were tested using the Chi-square test, with significance set at *p* < 0.05.

**Results:**

Although 60% of participants with a “good” body fat percentage demonstrated better performance, no statistically significant association was observed between body composition and swim time (*p* = 0.073). In contrast, participants who consumed carbohydrates in amounts exceeding ISSN recommendations before training showed significantly better performance (*p* = 0.003). Additionally, all participants whose pre-training carbohydrate intake was below the recommended levels demonstrated regular performance (*p* = 0.003).

**Conclusions:**

While body fat percentage is a relevant marker of body composition, this study suggests that adequate pre-training carbohydrate intake may have a greater impact on swimming performance. These findings reinforce the importance of carbohydrate availability as a key nutritional strategy, particularly in sports requiring both immediate and sustained energy, such as swimming.

